# Immunotherapy targeting cytokines in neuropathic pain

**DOI:** 10.3389/fphar.2013.00142

**Published:** 2013-11-22

**Authors:** Justin G. Lees, Samuel S. Duffy, Gila Moalem-Taylor

**Affiliations:** Department of Anatomy, School of Medical Sciences, University of New South WalesSydney, NSW, Australia

**Keywords:** neuropathic pain, cytokines, peripheral nerve injury, neuroimmunology, proinflammatory cytokines

Pain is a complex warning system activated in response to potential or apparent danger and the absence of pain is detrimental. Nociceptive pain is high-threshold pain activated in the presence of intense stimuli, such as contact with a burning object, and is a protective system essential for detection of noxious stimuli. Inflammatory pain, caused by immune system activation in response to tissue injury or infection, is also protective, as it discourages physical contact with the damaged area and assists healing (Woolf, 2010). In contrast, neuropathic pain emanating from disease or damage to the somatosensory system, is somewhat unique in that it is not protective, but rather pathological. Neuropathic pain encompasses a series of heterogeneous conditions with some similar clinical manifestations. Peripheral examples include traumatic nerve injury, diabetic peripheral neuropathy and chemotherapy-induced peripheral neuropathy, whilst multiple sclerosis is an example of a disease which can result in centrally derived neuropathic pain. These conditions are characterized by a low-threshold chronic pain emanating from aberrant peripheral and central neuronal sensitization. Symptoms include paraesthesia, spontaneous ongoing pain, and evoked pain (e.g., hyperalgesia and allodynia). Recent studies investigating neuropathic pain have demonstrated significant associated immune system activation and a fundamental role for cytokine signaling (Austin and Moalem-Taylor, 2010).

In this Opinion Article, we briefly summarize the progress made on research of cytokine involvement in neuropathic pain states and suggest that targeting key cytokines may prove useful in the development of new immune-therapeutics. However, further studies are required to determine which cytokine is the appropriate target for specific neuropathic pain conditions.

## Cytokine expression after nerve injury

Dysregulation of cytokines has been implicated in a variety of neuropathic pain conditions in both humans and animals. For example, differential expression of blood and cerebrospinal fluid cytokines has been demonstrated in patients with painful neuropathies. Compared to healthy controls, these patients show higher levels of proinflammatory cytokines [e.g., interleukin (IL)-1β and tumor necrosis factor-α (TNFα)], and lower levels of anti-inflammatory cytokines (e.g., IL-10) (Uceyler et al., 2007; Backonja et al., 2008). Animal models of peripheral nerve injury, such as chronic constriction injury (CCI) of the sciatic nerve, demonstrate extensive infiltration into the peripheral nervous system by cytokine producing immune cells (Moalem et al., 2004; Hu et al., 2007). Nerve injury increases expression and secretion of proinflammatory cytokines, including TNFα, IL-1β, IL-6, and interferon-γ, all of which are required for the development of pain hypersensitivity (Murphy et al., 1995; Costigan et al., 2009). Injury to neurons of the central nervous system (CNS) also initiates an immune response involving cytokine signaling (Guptarak et al., 2013).

In cases of peripheral nerve injury, the local inflammatory response is followed by a proximal response in both the dorsal root ganglion (DRG) and the spinal cord. Activated glial cells within the dorsal horn are critically involved in transmission of pain and are pivotal to the maintenance of chronic neuropathic pain (Austin and Moalem-Taylor, 2010). Both the major types of glia within the CNS (astrocytes and microglia) are particularly sensitive to activation following nerve injury (Mika et al., 2013). Furthermore, glia secrete cytokines and are likely to be the major source of central proinflammatory cytokines TNFα, IL-1β, and IL-6 (Whitehead et al., 2010). Classically characterized as messengers of the immune system, cytokines can act upon many different cell types, including neurons. For example IL-1β (Binshtok et al., 2008), TNFα (Jin and Gereau, 2006), and IL-17A (Richter et al., 2012) all can directly activate nociceptors and induce pain hypersensitivity.

Shortly after CCI, mRNA coding for the cytokine TNFα is rapidly elevated in the sciatic nerve (within hours of injury) and subsequently in the DRG (1–3 days following injury) (Sacerdote et al., 2008). TNFα alters the excitability of neurons and promotes continued inflammation (Sorkin et al., 1997). For at least 2 weeks, TNFα and its receptor TNFR1, display elevated expression in the ipsilateral and contralateral DRG of nerve-injured animals (Dubovy et al., 2006). Within the DRG and spinal cord, signaling via TNFR1, results in activation of NF-κ B pathway followed by up-regulation of IL-6 (Lee et al., 2009). Bilateral elevated levels of IL-6 are observed in the DRG following unilateral CCI and furthermore in distant cervical DRG, suggesting a wider neuroinflammatory response (Dubovy et al., 2013). IL-1β precipitates pain hypersensitivity when administered to the sciatic nerve (Zelenka et al., 2005) or intrathecally (Sung et al., 2004). *In vitro* exposure to IL-1β leads to increased excitability in medium and small DRG neurons (Stemkowski and Smith, 2012). IL-1β also acts in higher CNS regions whereby supraspinal changes in IL-1β expression alter over time in the brainstem, thalamus and prefrontal cortex following sciatic nerve injury (Apkarian et al., 2006). Furthermore, IL-1β is elevated in the contralateral hippocampus of rats with CCI and spinal nerve ligation (SNL) (del Rey et al., 2011).

## Therapeutics targeting proinflammatory cytokines

Numerous studies have demonstrated that antagonism of proinflammatory cytokine signaling attenuates neuronal hypersensitivity and inflammation associated with nerve injury. For example, intrathecal injection of both IL-1β and TNFα antagonists alleviated pain induced by gp120 activated spinal injury (Milligan et al., 2001). Similarly, intrathecal administration of an IL-6 neutralizing antibody significantly reduced gp120-induced mechanical allodynia and down-regulated the expression of IL-1β and TNFα within the CNS (Schoeniger-Skinner et al., 2007). Biologics which target the activity of specific proinflammatory cytokines have been and continue to be developed. A common theme associated with their clinical use is that they are generally well-tolerated (Dinarello et al., 2012).

### TNFα inhibitors

TNFα inhibitors are demonstrated to significantly reduce mechanical and thermal pain hypersensitivity associated with peripheral nerve injury (Iwatsuki et al., 2013). There are several TNFα inhibitors which have been developed, including the humanized monoclonal antibody infliximab and the receptor fusion protein etanercept. These and other TNFα inhibitors are currently approved for clinical use to treat a range of immune disorders including rheumatoid arthritis, chrohn's disease, and psoriasis. Infliximab was tested in a human clinical trial for the treatment of pain associated with disc herniation induced sciatica. The drug failed to show a significant reduction in pain across the entire treatment group, but patients with L3–L4 and L4–L5 disc herniation appeared to benefit (Korhonen et al., 2006). Etanercept was tested in humans suffering from sciatica and shown to have significant benefits in a pilot study (Genevay et al., 2004). Similarly in another small trial, epidural administration of etanercept was effective in reducing pain associated with spinal stenosis (Ohtori et al., 2012a). However, results from a randomized, double blind, placebo controlled trial for the treatment of radicular or discogenic back pain indicated that etanercept did not demonstrate any significant benefit (Cohen et al., 2007). TNFα activates the pain mediator p38 following nerve injury, a signaling pathway which is thought to be critical for the mediation of pain transmission. Pre-treatment with inhibitors of p38 (SB203580) or etanercept significantly reduced mechanical allodynia in rats with SNL injury (Schafers et al., 2003). In a human clinical trial of patients with nerve trauma, radiculopathy, or carpal tunnel syndrome, the selective p38 inhibitor dilmapimod was shown to have a statistically significant effect in reducing patients pain scores (Anand et al., 2011). Conversely, a similar p38 selective inhibitor losmapimod did not show any significant effect in reducing pain in a human clinical trial of patients with peripheral focal neuropathic pain related to nerve injury caused by trauma or surgery (Ostenfeld et al., 2013). Despite some variable clinical trial outcomes, there is evidence to suggest that TNFα inhibitors and next generation p38 inhibitors may prove effective in the treatment of different forms of neuropathic pain.

### IL-1β inhibitors

Both knockout of the IL-1 receptor and transgenic over expression of the endogenous receptor antagonist IL-1RA reduced mechanical and thermal pain hypersensitivity in mice with spinal nerve injury (Wolf et al., 2006). Similarly, a neutralizing antibody targeting the IL-1β receptor (IL-1R1) alleviated allodynia in mice with CCI (Sommer et al., 1999). Therapeutics targeting the IL-1 receptor such as anakinra, a recombinant form of IL-1RA, the soluble decoy receptor rilonacept and anti IL-1β neutralizing antibody cannikumab, are all currently approved for use in a number of inflammatory diseases, including rheumatoid arthritis, gout, and stills diseases. A current clinical trial is testing whether preoperative administration of anakinra reduces incisional pain in patients undergoing vascular or orthopedic surgical procedures by lowering the concentration of inflammatory mediators in surgical wounds (NCT01466764). Given the key role of IL-1β in the development and propagation of neuropathic pain in animal models, it is noteworthy that very few trials have been conducted testing the effects of IL-1β inhibitors against neuropathic pain conditions in humans.

### IL-6 inhibitors

IL-6 is widely expressed following nerve injury and intrathecal administration of IL-6 results in mechanical allodynia. An IL-6 receptor neutralizing antibody abolished mechanical allodynia associated with spinal cord injury pain 14 days after a single intraperitoneal injection (Guptarak et al., 2013). The humanized IL-6 receptor neutralizing antibody tocilizimab has been approved for use in rheumatoid arthritis and juvenile idiopathic arthritis. There have also been case reports where this antibody has been effective in reducing pain associated with neuromyelitis optica (Araki et al., 2013) and sciatica (Ohtori et al., 2012b). Alternative antibodies that bind directly to IL-6 (BMS945439) are being tested in clinical trials for the treatment of rheumatoid arthritis. Due to the substantial evidence indicating a role for IL-6 in the propagation of neuropathic pain, therapeutic antibodies targeting both the IL-6 and its receptor may be beneficial in treating certain neuropathic pain conditions.

### IL-17

Although less conspicuous than other proinflammatory cytokines, IL-17 is a key orchestrator of cytokine signaling in neuropathic pain. IL-17 expression is significantly elevated following CCI in mice, peaking at day 7 after injury (Kleinschnitz et al., 2006). Knockout of IL-17 reduces pain hypersensitivity and the activation of CNS glia when compared to wild type mice following partial sciatic nerve ligation (PSNL), conversely injection of IL-17 results in increased mechanical and thermal hypersensitivity and neutrophil migration to the site of injection (Kim and Moalem-Taylor, 2011). IL-17 mediated hyperalgesia has been shown to be dependent on TNFα/TNFRA1 signaling (McNamee et al., 2011). Intraperitoneal injection of a monoclonal antibody against IL-17 into rats with antigen induced arthritis reduced paw guarding and hyperalgesia (Richter et al., 2012). Humanized monoclonal antibodies against IL-17, such as secukinumab, are now being assessed for efficacy in treating a range of inflammatory conditions including psoriasis and rheumatoid arthritis, and based on results with animal models these biologics could be tested in human clinical trials for treatment of neuropathic pain.

## Therapeutics aimed at resolving inflammation

As an alternative to targeting proinflammatory cytokines, another treatment option is to promote resolution of the inflammation by stimulating the expression of anti-inflammatory cytokines. A perceived advantage of this strategy is that it does not directly inhibit the activity of proinflammatory cytokines, which may be required for processes such as Wallerian degeneration and peripheral axonal regeneration.

### Anti-inflammatory cytokines

A single dose of IL-10 had long-lasting behavioral effects in rats with excitotoxic spinal cord injury (Plunkett et al., 2001). Intrathecal gene therapy targeting the expression of IL-10 has been shown to be efficacious; delivery of adeno-associated viral IL-10 transiently reversed allodynia (Milligan et al., 2005), whilst repeated delivery of naked DNA encoding IL-10 reversed allodynia for up to 2 months after CCI (Milligan et al., 2006). Administration of IL-4 inhibited the production of TNFα and IL-1β in hyperalgesia models (Cunha et al., 1999) and intraneural injection of TGFβ caused a delayed and reduced pain hypersensitivity in rats with PSNL. This attenuated the homing of cytokine producing MAC+ macrophages and reduced the infiltration of T cells into the injured nerve (Echeverry et al., 2013). Data on the effectiveness of anti-inflammatory therapies in treating neuropathic pain in humans is limited, and has to date focused largely on IL-10. Recombinant human IL-10 (such as Ilodecakin/Tenovil) has been tested with variable success in treating chronic inflammatory conditions such as psoriasis, Crohn's disease, and rheumatoid arthritis. To the best of our knowledge, human trials investigating the efficacy of similar therapies in treating neuropathic pain are yet to be conducted.

## Summary and perspective

The remarkable success of targeted inhibition of several cytokines, such as TNFα, in patients with rheumatoid arthritis, psoriasis and many other diseases has fundamentally revised the treatment of chronic inflammatory diseases. This suggests that different conditions may share common pathophysiology and may benefit from disruption of the cytokine network. Indeed, several neuropathic pain conditions have been shown to have dysregulation of cytokines, and the use of biologics targeting cytokines is an exciting and promising strategy in the quest to find more effective treatments for neuropathic pain. There are two basic sub-strategies that can be followed: (1) to target certain proinflammatory cytokines or their receptors to inhibit their activity, (2) to enhance the resolution of inflammation by promoting the activity of anti-inflammatory cytokines. The few clinical trials that have tested the efficacy of cytokine inhibitors in chronic neuropathic pain so far have demonstrated mixed results, suggesting that human validation studies will be necessary to identify the appropriate cytokines for a given neuropathic pain syndrome. It is anticipated that new effective cytokine targets will be discovered and will allow future novel treatment strategies for neuropathic pain.
